# Assessing causal relationship between obesity and rosacea: A two‐sample Mendelian randomization study

**DOI:** 10.1111/jocd.16392

**Published:** 2024-05-20

**Authors:** Huiyi Yao, Sihao Shen, Xingyue Gao, Xiuzu Song, Wenzhong Xiang

**Affiliations:** ^1^ Department of Dermatology, Hangzhou Third People's Hospital Zhejiang Chinese Medical University Hangzhou China; ^2^ Department of Dermatology, Hangzhou Third People's Hospital Hangzhou Third People's Hospital Affiliated to Zhejiang Chinese Medical University Hangzhou China

To the Editor,

Rosacea is a chronic inflammatory skin disorder that is distinguished by recurrent or persistent central facial flushing and erythema.^1^ As a major global health concern, obesity is defined by body mass index (BMI) ≥ 25 kg/m^2^.^2^ Observational studies have reported mixed results regarding the association between obesity and rosacea. Thus, whether the two are causally associated remains unclear. Mendelian randomization (MR) is an effective method for determining causality and reducing confounding biases, which is based on the theory of random assignment of alleles and related genome‐wide association studies (GWAS).^3^ Our study aimed to explore the causal relationship between obesity and rosacea using a two‐sample MR study.

The FinnGen database (http://www.finngen.fi) was used to retrieve the GWAS data for rosacea (1195 cases and 211 139 controls), which included both males and females in the European population. For exposures, we retrieved BMI, waist circumference, hip circumference, waist–hip ratio (WHR), and obesity from GWAS summary data sets at http://app.mrbase.org/, limiting the population to “European” and sex to “Males and Females” for conformance with the rosacea data set. The *p*‐values of SNPs below 5 × 10^−8^ were selected, and we found two data sets of WHR (ID: ieu‐a‐87, ieu‐b‐4829) in which the number of SNPs was less than 3 in the MR analysis. Therefore, we adjusted the *P*‐values of the SNPs of these two data sets to be below 5 × 10^−6^. Meanwhile, *r* ^2^ < 0.001 and 10 000 kb were set to reduce interference from linkage disequilibrium (LD). Additionally, F‐statistics were also calculated using the formula $F=\left(\frac{n-k-1}{k}\right)\left(\frac{R^{2}}{1-R^{2}}\right)$, in which a value of more than 10 suggested that the strength of the SNPs was adequate.^4^ Moreover, the formula $F=\frac{\beta^{2}}{{\mathrm{SE}}^{2}}$ ^5^ was implemented in those data sets missing the eaf values (effect allele frequency). Inverse‐variance weighted (IVW) was performed as the primary MR analysis method. To further enhance reliability, MR‐Egger and weighted median analyses were also performed. Furthermore, Cochran's *Q* test and MR‐Egger Intercept were applied as sensitivity analyses.

In total, eight data sets on BMI, four on waist circumference, four on hip circumference, five on WHR, and one on obesity were included. The results of the MR analysis showed that all *p*‐values were >0.05. The odds ratio (OR) and 95% confidence interval (CI) enhanced the validity of our findings (Figure 1). Every SNP exhibited strong robustness, as evidenced by all F‐statistics of >26 (Table S1). These outcomes were confirmed using sensitivity analyses (Table 1). Our results demonstrated no association between genetic susceptibility to obesity and a risk of rosacea.

**FIGURE 1:**
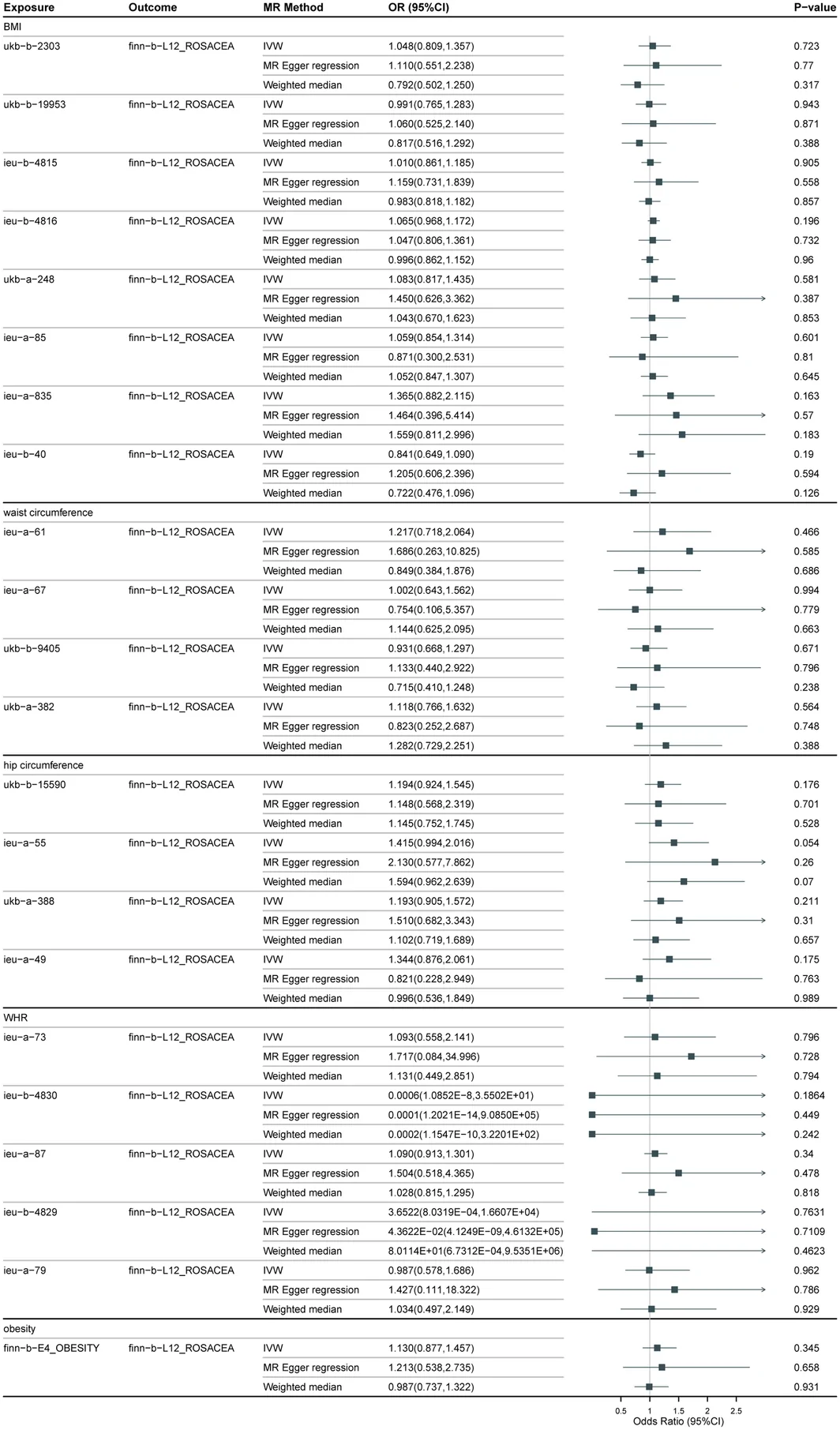
MR analysis results. Applying *p*‐values, odds ratios, and 95% confidence intervals to estimate whether obesity has a causal effect on rosacea. BMI, body mass index; CI, confidence intervals; IVW, inverse‐variance weighted; MR, Mendelian randomization; OR, odds ratio; WHR, waist–hip ratio.

**TABLE 1:** Sensitivity analyses of obesity and the risk of rosacea.

Exposures		Outcome	SNPs	Heterogeneity test		MR Egger pleiotropy test	
Name	ID			Q	P‐value	Intercept	P‐value
BMI	ukb‐b‐2303	Rosacea	424	444.6349	0.2253	−0.0011	0.8620
	ukb‐b‐19 953	Rosacea	432	451.4901	0.2389	−0.0013	0.8395
	ieu‐b‐4815	Rosacea	7	7.6892	0.2618	−0.0387	0.5569
	ieu‐b‐4816	Rosacea	35	31.4232	0.5946	0.0031	0.8918
	ukb‐a‐248	Rosacea	297	337.6422	0.0480	−0.0064	0.4700
	ieu‐a‐85	Rosacea	7	11.0078	0.0881	0.0486	0.7283
	ieu‐a‐835	Rosacea	67	70.0689	0.3428	−0.0020	0.9117
	ieu‐b‐40	Rosacea	481	468.1138	0.6425	−0.0063	0.2677
WC	ieu‐a‐61	Rosacea	40	35.6919	0.6216	−0.0091	0.7220
	ieu‐a‐67	Rosacea	63	50.3234	0.8558	0.0075	0.7719
	ukb‐b‐9405	Rosacea	351	376.0759	0.1618	−0.0033	0.6630
	ukb‐a‐382	Rosacea	221	263.4131	0.0239	0.0058	0.5931
HC	ukb‐b‐15 590	Rosacea	398	386.7799	0.6339	0.0008	0.9054
	ieu‐a‐55	Rosacea	73	59.9037	0.8448	−0.0128	0.5259
	ukb‐a‐388	Rosacea	279	295.3972	0.2263	−0.0053	0.5347
	ieu‐a‐49	Rosacea	51	43.0354	0.7467	0.0157	0.4264
WHR	ieu‐a‐73	Rosacea	28	16.4215	0.9444	−0.0118	0.7656
	ieu‐b‐4830	Rosacea	13	5.3131	0.9467	0.0051	0.8652
	ieu‐a‐87^a^	Rosacea	9	5.9261	0.6555	−0.0550	0.5674
	ieu‐b‐4829*	Rosacea	14	7.2722	0.8876	0.0198	0.5415
	ieu‐a‐79	Rosacea	36	19.5158	0.9840	−0.0107	0.7738
Obesity	finn‐b‐E4_ OBESITY	Rosacea	8	8.4333	0.2959	−0.0117	0.8629

Abbreviations: BMI, body mass index; HC, hip circumference; SNPs, number of single nucleotide polymorphism; WC, waist circumference; WHR, waist–hip ratio.

^a^ *p*‐Values of the SNPs of these two data sets were below 5 × 10^−6^, others were below 5 × 10^−8^.

Our findings are contrary to some observational studies. This discrepancy may occur not only because measurement errors in observational studies limit the ability to deduce causality and disclose associations without direction,^3^ but also because of the nonextensiveness of populations in each study. A retrospective study^6^ only enrolled nasal rosacea patients and showed a positive relationship between obesity and rosacea, suggesting that different subtypes and areas of rosacea may result in different outcomes. Furthermore, Wang et al.^2^ revealed that although there was no significant correlation between body weight status alone and rosacea, the interaction between body weight and consumption of spicy had an impact on erythematotelangiectatic and severe rosacea. This serves as a clinical guideline for further research on diet and weight control in rosacea patients. The limitations of this study were that the data sets only recruited Europeans, and we did not study the effect of obesity on different subtypes and areas of rosacea since we did not find a data set that categorized rosacea.

In conclusion, our study demonstrated that there is no association between obesity and rosacea. Further research is required to confirm whether the conclusions can be generalized to all races as well as subtypes and regions of rosacea.

## AUTHOR CONTRIBUTIONS

**Huiyi Yao**: Conceptualization, Methodology, Formal analysis, Resources, Writing—Original draft Preparation and Review and Editing. **Sihao Shen**: Methodology, Formal analysis, Resources, Writing—Review and Editing. **Xingyue Gao**: Formal analysis, Data curation, Writing—Review and Editing. **Xiuzu Song**: Formal analysis, Investigation, Writing—Review and Editing. **Wenzhong Xiang**: Formal analysis, Investigation, Writing—Review and Editing. Final approval of the version to be published: All authors. All authors agree to be accountable for all aspects of the work.

## FUNDING INFORMATION

This work was supported by the Hangzhou Biomedical and Health Industry Development Support Project (2021WJCY159) and Hangzhou Medical Key Discipline Construction Project (no. [37]21‐3).

## CONFLICT OF INTEREST STATEMENT

The authors report no relationships that could be construed as a conflict of interest.

## Supporting information


Table S1.

## Data Availability

The data that support the findings of this study are available from the corresponding author upon reasonable request.
