# Supplementary material for: Assessing causal relationship between obesity and rosacea: A two‐sample Mendelian randomization study
Source: J Cosmet Dermatol. 2024 May 20;23(10):3421–4. doi: 10.1111/jocd.16392 (PMC13648888; doi:10.1111/jocd.16392)
Supplement: Supplementary file 1 [file JOCD-23--s001.docx]

**Table S1. Information of exposures**

| **Exposures** | | **Sex** | **Population** | **nSNP^a^** | **Sample size** | **Total R^2^** | **F-statistics** |
| --- | --- | --- | --- | --- | --- | --- | --- |
| **Name** | **ID** |  |  |  |  |  |  |
| BMI | ukb-b-2303 | Males and Females | European | 448 | 454884 | 0.0569 | 61.2 |
|  | ukb-b-19953 | Males and Females | European | 458 | 461460 | 0.0573 | 61.2 |
|  | ieu-b-4815 | Males and Females | European | 11 | 51852 | NA | 61.4 |
|  | ieu-b-4816 | Males and Females | European | 42 | 99998 | NA | 56.8 |
|  | ukb-a-248 | Males and Females | European | 315 | 336107 | 0.0519 | 58.4 |
|  | ieu-a-85 | Males and Females | European | 7 | 16068 | 0.1401 | 373.9 |
|  | ieu-a-835 | Males and Females | European | 69 | 322154 | 0.0227 | 106.8 |
|  | ieu-b-40 | Males and Females | European | 507 | 681275 | 0.0526 | 74.5 |
| WC | ieu-a-61 | Males and Females | European | 42 | 232101 | 0.0144 | 80.8 |
|  | ieu-a-67 | Males and Females | European | 64 | 231353 | 0.0183 | 66.3 |
|  | ukb-b-9405 | Males and Females | European | 374 | 462166 | 0.0345 | 44.1 |
|  | ukb-a-382 | Males and Females | European | 232 | 336639 | 0.0292 | 43.7 |
| HC | ukb-b-15590 | Males and Females | European | 420 | 462117 | 0.0536 | 62.2 |
|  | ieu-a-55 | Males and Females | European | 75 | 211114 | 0.0275 | 79.6 |
|  | ukb-a-388 | Males and Females | European | 285 | 336601 | 0.0476 | 59.0 |
|  | ieu-a-49 | Males and Females | European | 52 | 213038 | 0.0183 | 76.2 |
| WHR | ieu-a-73 | Males and Females | European | 29 | 212244 | 0.0078 | 57.4 |
|  | ieu-b-4830 | Males and Females | European | 16 | 85978 | NA | 54.2 |
|  | ieu-a-87^b^ | Males and Females | European | 9 | 10255 | 0.1142 | 146.8 |
|  | ieu-b-4829^b^ | Males and Females | European | 16 | 31740 | NA | 26.3 |
|  | ieu-a-79 | Males and Females | European | 38 | 210082 | 0.0119 | 66.5 |
| obesity | finn-b-E4_ OBESITY | Males and Females | European | 8 | 218735 | 0.0621 | 1810.5 |

Abbreviations: nSNPs, Number of single nucleotide polymorphism; BMI: body mass index; WC: waist circumference; HC: hip circumference; WHR, waist-hip ratio; NA: not available.

^a^: Number of SNPs of exposures that meet the correlation settings.

^b^: p-values of the SNPs of these two datasets were below 5×10^−6^, others were below 5×10^−8^.
